# The Clinical Efficacy of Minimally Invasive Clamp-Assisted Reduction and Open Reduction with Wire Cerclage for Unstable Subtrochanteric Fractures

**DOI:** 10.1155/2022/5340504

**Published:** 2022-01-25

**Authors:** Dong Liu, Hong-zhi Liu, Ming-liang Ma, Nan Zhou, Hui Wang

**Affiliations:** ^1^Department of Orthopedics Trauma, Binzhou Medical University Hospital, Binzhou, Shandong, China; ^2^Department of Trauma Center, Binzhou Medical University Hospital, Binzhou, Shandong, China; ^3^Department of Orthopedics, The First Affiliated Hospital of Guangxi Medical University, Nanning, Guangxi, China

## Abstract

The purpose of this study was to compare the clinical effectiveness of minimally invasive clamp-assisted reduction and open reduction with wire cerclage and intramedullary nails for unstable subtrochanteric fractures. Between January 2016 and October 2019, 68 patients who had unstable subtrochanteric fractures experienced intramedullary nail surgery in this retrospective study. There were 41 cases in the minimally invasive clamp or closed reduction group (group A) and 27 cases in the open reduction with wire cerclage group (group B). There were 3 cases of complications in group A and 2 cases of complications in group B. Remarkable distinction was observed between the two groups in the operation time (*p* < 0.05), quality of reduction (*p* < 0.05), and union time (*p* < 0.05). For the successful surgical treatment of unstable femoral subtrochanteric fractures, an anatomical reduction is crucial. Reduction and wire cerclage are cut to give medial support for the anatomical reduction, which has a positive effect on fracture healing.

## 1. Introduction

About 5% to 20% of proximal femoral fractures are femoral subtrochanteric fractures [1]. The area expanding 5 cm from the distal end of the lower edge of the lesser trochanter is the subtrochanteric area [2]. The disease is mainly manifested as a bimodal distribution. For young patients, high-energy trauma caused subtrochanteric fractures. For elderly patients, low-energy traumas, such as falls, cause subtrochanteric fractures [3]. Because of concentrated stress and variables in this fractured area, after a fracture, the proximal end of the fracture exhibits flexion, abduction, and external rotation displacement caused by traction from the gluteus muscle, iliopsoas muscle, and the external rotator muscle group [4]. Hence, surgery is usually required. Intramedullary central fixation is mechanically and biologically advantageous, and hence, it is the preferred choice for the treatment of subtrochanteric fractures [5, 6].

Based on the existing research, the key to good results in subtrochanteric fractures is proper reduction [4–7]. These fractures are challenging to treat, even for experienced fracture surgeons. In the nailing process, many skills and methods were come up with to keep the reduction. The most common techniques are clamp-assisted reduction and cerclage wire reduction [8]. As closed reduction is sometimes impossible to provide satisfactory results, open reduction is necessary. More people apply wire or cerclage for fracture reduction and fixation after incision [9]. By anatomically reducing the fracture and propping up the medial hinge, the open reduction of these fractures is facilitated by the cerclage wire [10]. The advantages of cerclage wire in the treatment of subtrochanteric fractures have been reported by some studies because they can keep the fracture reduction without affecting the intramedullary nail operation. It is suggested to do it with as diminutive additional soft tissue damage and as few wires as possible [11, 12]. Its use was also favored by biomechanical studies since it guaranteed the medial cortical stent could be repositioned. A steadier configuration was gained, and the fixation failure risk was reduced. There are other studies that encourage biological fixation and discourage the use of cerclage wires because they disrupt the estimated blood supply [13–21].

Minimally invasive clamp-assisted reduction for refractory intertrochanteric fracture is simple, effective, and less traumatic. For the refractory intertrochanteric fracture of the femur associated with the external wall displacement, the external wall should be reinforced after clamping reduction and intramedullary nail fixation to avoid reduction loss and internal fixation failure. Dr. T. Apivatthakakul of the Department of Orthopedics, Chiang Mai University School of Medicine, Thailand, has designed a percutaneous wire annulus technique called open reduction with wire cerclage to assist reduction and fixation of fractures. In a paper published in Injury, the authors point out that the technique could be an alternative to the surgical treatment of complex fractures by reducing the surgeon's radiation exposure. To compare the two different ways of surgery, the following outcomes were used: tip-apex distance (TAD), varus/valgus, Harris hip score (HHS), and quality of reduction. Other measures include blood loss during the operation, the operation time, fracture healing time, hospital stay after surgery, follow-up time, and complications.

The specific contributions of this paper include: (1) this study showed that the vital point to the successful treatment of unstable femoral subtrochanteric fractures was the anatomical reduction, and the use of cerclage wires may improve the fracture reduction effect, which is the first study that compares the clinical efficacy of minimally invasive clamp-assisted reduction and open reduction with wire cerclage for unstable subtrochanteric fractures. (2) Our results indicated that the benefits of using cerclage wire for fracture reduction outweigh the risks, although many subtrochanteric fractures may be successfully treated with a single indirect reduction.

The rest of this paper is organized as follows: between January 2016 and October 2019, 68 patients who had unstable subtrochanteric fractures experienced intramedullary nail surgery in this retrospective study. There were 41 cases in group A and 27 cases in group B. The classification was based on Seinsheimer criteria. Minimally invasive clamp or closed reduction was adopted by group A. Group B was treated with open reduction with wire cerclage. Each patient was followed up for 12 to 31 months.

In this study, 68 cases of unstable subtrochanteric fractures that received a minimally invasive clamp-assisted reduction or open reduction with wire cerclage were estimated. The purpose of the study was to evaluate the effect of different reduction methods on the stability of subtrochanteric fractures and to evaluate the patients' clinical results.

## 2. Methods and Materials

### 2.1. Patients

**Figure 1:**
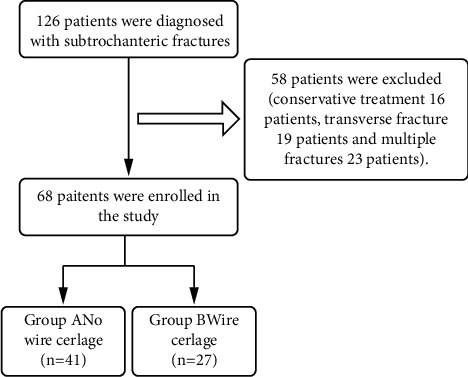
Study flowchart.

The ethics committee of the Affiliated Hospital of Binzhou Medical College approved this retrospective study. Between January 2016 and October 2019, a total of 126 patients with subtrochanteric fractures were initially involved in the study. There are two aspects to fractures: the fractures in the subtrochanteric area and the reverse oblique fractures that enlarge to the subtrochanteric area. The inclusion standards were as follows: ① unstable subtrochanteric fractures; ② no severe cognitive impairment; ③ at least 1 year of follow-up; ④can walk before fracture independently. The exclusion criteria were as follows: ① patients with conservative treatment; ② transverse fractures; ③ multiple fractures. Finally, this study included a total of 68 patients (the study flow diagram is shown in Figure 1), and they were divided into two groups: group A and group B. Group A was treated with minimally invasive clamp-assisted reduction, and group B was treated with open reduction with wire cerclage. Group A consisted of 41 patients, aged from 31 to 83 years, including 30 males and 11 females. Group B consisted of 27 patients, aged from 30 to 83 years, including 19 males and 8 females. Based on the classification standard named Seinsheimer [14], group A had 13 type II b cases, 11 type III a cases, 2 type II c, 5 type V cases, 1 case of III b, and 9 type IVcases. Group B had 7 type IV cases, 8 type II b cases, 1 type II c case, 4 type III a cases, 5 type V cases, and 2 III b cases.

### 2.2. Preoperative Treatment

After hospitalization, the patients in both groups were treated with tibial tubercle bone traction (hip flexion and knee flexion abduction position, with a traction weight of 1/8 of body weight), and the thigh muscle was fully relaxed. The fracture was reduced, and a soft tissue hinge was used to maintain the alignment of the fracture end to facilitate fracture reduction during the operation. Rivasaban was taken orally before the operation to prevent venous thrombosis of the lower extremities.

### 2.3. Surgical Procedure

#### 2.3.1. Anesthesia and Position

**Figure 2:**
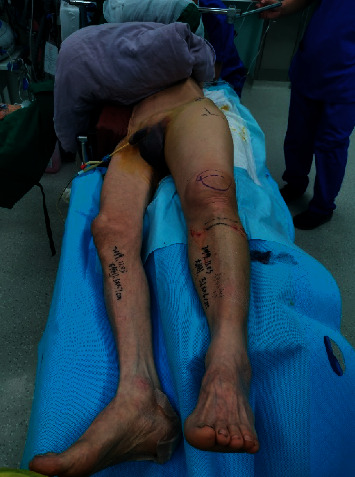
The patient took the half-side lying position. The affected side was cushioned high with a soft pad, which was at an angle of about 30 with the horizontal direction, and the healthy side was protected by a bed block to prevent falling.

Epidural anesthesia or general anesthesia was performed during the operation, and the affected side was placed in the lateral recumbent position, i.e., the buttocks and lower limbs were raised by approximately 30° from the operating table (shown in Figure 2) so that positive and lateral fluoroscopy could be performed easily during the operation.

#### 2.3.2. Installation of Lower Limb Retractor

**Figure 3:**
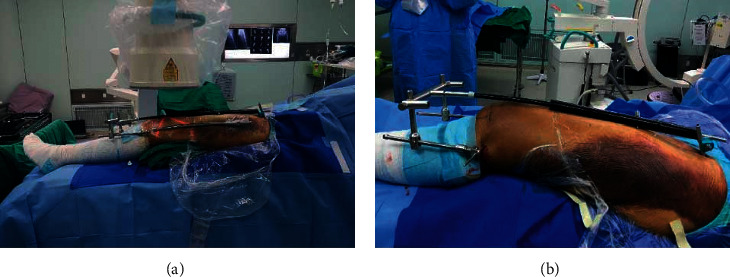
Schematic diagram of the installation of lower limb retractor. Firstly, a 4 mm Kirschner wire is placed in the anterior inferior spine. Then, a 4 mm Kirschner wire is inserted across the middle and back of the distal femur, and the middle part is connected with a carbon rod. The fracture is reduced by rotating the internal and external screws of the distal femur with a wrench.

As shown in Figure 3, after the operation area was disinfected and an aseptic sheet was laid, traction reduction was performed with a lower limb retractor. The installation process is as follows: ① the anterior inferior spine on the body surface is located, a 4 mm Kirschner wire and a cannula are placed at that location; ② a 4 mm Kirschner wire is crossed with the middle and posterior part of the femoral condyle; ③ an extension rod is installed, the distal and proximal Kirschner wires are connected; ④ traction works on the end of the broken fracture for reducing the fracture.

#### 2.3.3. Reduction and Fixation of Group A

After the fracture length was restored by traction, if there was still rotation or angular displacement at the fracture end, the fractures in group A were reduced by prying and pushing the top with a Kirschner wire, the failed reduction was assisted by a limited open clamp, and fluoroscopy was used to visualize the end of the broken fracture. When the reduction extent was considered satisfactory, the guide needle was placed through the top of the greater trochanter. When it was confirmed that the guide needle was in the medullary cavity center of the proximal femur, the proximal and distal medullary cavities of the femur were enlarged with a dynamic reaming drill, the main femoral intramedullary nail was placed, and finally, the proximal and distal intramedullary nails were locked sequentially.

#### 2.3.4. Reduction and Fixation of Group B

The patients in group B were healed by open reduction and encircling fixation with steel wires for 1–2 channels. Before the operation, the incision range was determined by X-ray fluoroscopy according to the fracture line, and the steel wire or titanium cable was minimally inserted into the wire or titanium cable before reaming. The follow-up operation was the same as that performed for group A.

### 2.4. Postoperative Treatment

Anticoagulation therapy was continued after the operation, and the patient underwent passive movement of the lower extremity joints on the 2^nd^ day after the operation. After the end of treatment, the details of the operation, clinical efficacy, and complications were recorded and evaluated for both groups of patients.

### 2.5. Outcome Measures

#### 2.5.1. Tip-Apex Distance (TAD)

The distance from the lag screw tip to the femoral head tip on the AP and lateral view is TAD, measured in millimeters. When TAD < 25 mm, the slightest risk is to use a lag screw to cut the femoral head upward.

#### 2.5.2. Varus/Valgus

The normal range of the cervical shaft angles was 110° to 140°, with an average of 127°. Hip varus is defined as hip varus if the angle is less than the normal range, and hip valgus is if the angle is greater than the normal range.

#### 2.5.3. Harris Hip Score (HHS)

To assess the adults' recovery of hip joint function, HHS was applied. Mainly, the HHS scoring system involves 4 fields, including function, pain, deformity absence, and motion range. 100 points are the maximum (best possible outcome). Poor performance is a total score of <70. The fair performance is 70–80. The good performance is 80–90. The excellent performance is 90–100.

#### 2.5.4. Quality of Reduction

Based on the good, acceptable, or poor classification law of Baumgartner et al., the reduction quality was estimated [15]. According to the X-ray immediately after the operation and the displacement and alignment seen, the classification was evaluated. The AP X-ray showed normal or tiny valgus alignment to categorize the reduction as good. The outside angle was less than 20°, and the fragments were not displaced by more than 4 mm. One or two standards can only be met by an acceptable reduction, but not all. No standard would be met by a poor reduction.

#### 2.5.5. Other Measures

Other measures include blood loss during the operation, the operation time, fracture healing time, hospital stay after surgery, follow-up time, and complications. The two groups were compared.

### 2.6. Statistical Analysis

The study used SPSS Version 24 to analyze the data (SPSS, Chicago, Illinois, USA). Also, we used the Mann–Whitney U exam or Students' test to contrast the time of operation, the estimated blood loss, TAD, varus/valgus, union time, and HHS between the two groups. We applied the chi-square exam or Fisher's exact exam to contrast age, sex, the Seinsheimer classification, the type of implant, complications, and quality of reduction between the two groups. If *p* ≤ 0.05, there is a significant statistical difference.

## 3. Results

### 3.1. Follow-Up

Follow-up time was at least one year. After surgery, X-ray examinations were performed after 3, 6, and 12 months. HHS was also performed during each follow-up.

### 3.2. General Results

As shown in Table 1, in terms of gender, age, implant type, fracture type, intraoperative blood loss, etc., the two groups of patients had no significant differences (*p* > 0.05). However, the operation time was 102.05 ± 29.04 min in group A and 124.01 ± 35.28 minutes in group B. There was a significant difference between the two groups (*p*=0.04) (Shown in Table 1).

### 3.3. Quality of Reduction

Compared with group A, as expected, the group B quality reduction is remarkably better in Table 1.

This makes sense in statistics (*p*=0.03). In group A (*n* = 41), the good reduction was achieved in 24 cases, acceptable reduction in 12 cases, and poor reduction in 5 cases. Good reduction accounted for 58.54% (24/41). In group B (*n* = 27), the good reduction was achieved in 22 cases, acceptable reduction in 5 cases, and there were no patients with poor reduction. Good reduction accounted for 81.48% (22/27).

### 3.4. Implants Evaluation

There were no significant differences in TAD and varus/valgus between the two groups. There was no significant difference in the imaging evaluation results of the two groups at 3 months and 6 months after surgery.

### 3.5. Functional Evaluation

**Figure 4:**
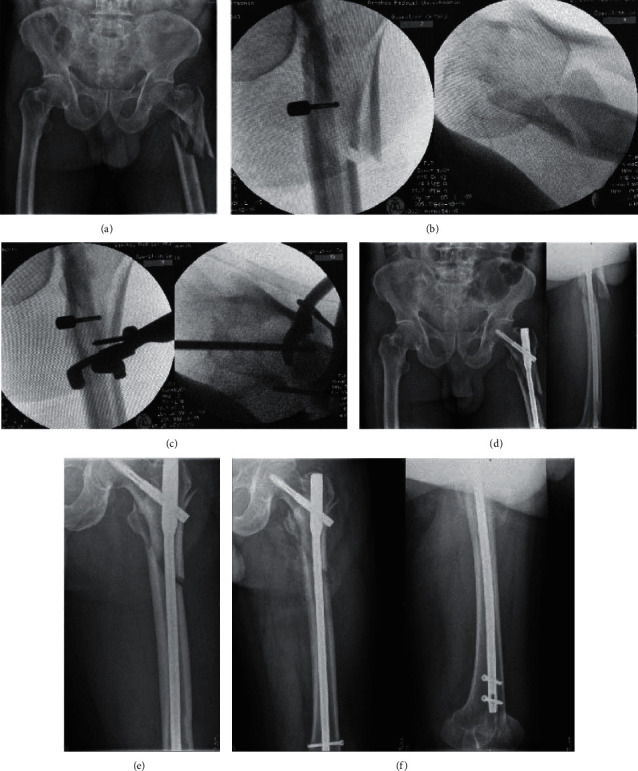
Preoperative X-ray of the patient, Seinsheime classification V (a). During the operation, the length of the fracture was restored by traction with the retractor, and the displacement of the fracture block was obvious (b). By the minimally invasive way, the reduction forceps are used to reduce the fracture piece from the front and the outside to achieve anatomical reduction as far as possible (c). X-ray examination was performed 2 days after surgery. It showed that the fracture piece was displaced, which proved that the fracture piece was unstable (d). Two months after the operation, it was found that there was no callus formation at the fracture site, and the fracture line was still clear (e). Six months after the operation, it was found that a large number of calluses formed at the end of the fracture, and the fracture healed well (f).

**Figure 5:**
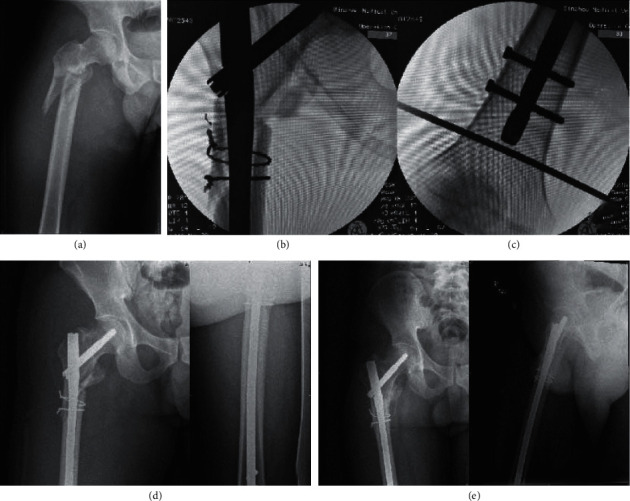
Preoperative X-ray of the patient, Seinsheime classification III a (a). During the operation, the length of the fracture was restored by traction with the retractor. Then, the broken end of the fracture was cut open, and the fracture was anatomically reduced, fixed with two wires, and fixed with a full-length intramedullary nail (b). X-ray examination was performed 2 days after surgery. It showed that the fracture had a good reduction, and there was no valgus deformity (c). Three months after the operation, the fracture line disappeared and healed well (d).

As shown in Figures 4 and 5, there were no significant differences in HHS (*p* > 0.05) between group A and group B. In group A, the patients' curing time was 4.82 ± 0.89 months, while in group B, the time was 3.56 ± 0.64 months. The difference between group A and group B is remarkable (*p*=0.01) (shown in Figures 4 and 5).

#### 3.5.1. Complications

**Figure 6:**
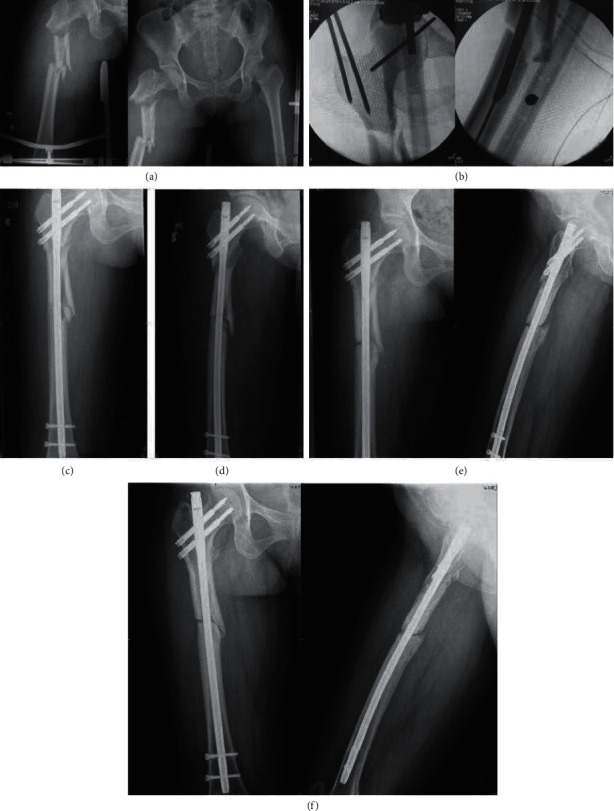
Preoperative X-ray of the patient, Seinsheime classification IV (a). During the operation, the length of the fracture was restored by traction with the retractor. Then, closed reduction was used during the operation. The gold finger was used to pass through the broken end of the fracture, and the full length intramedullary nail was fixed (b, c). Two months after the operation, the position of internal fixation was good, and there was no sign of fracture healing (d). Six months after the operation, X-ray examination showed that the proximal fracture line was blurred, and the distal fracture line was still clearly visible (e). Twelve months after the operation, X-ray examination showed that the proximal fracture line disappeared, the distal fracture line was clear, and the broken end of the fracture hardened and was diagnosed as atrophic nonunion (f).

Group A had 3 cases of complications, including 2 cases of bone nonunion and 1 case of superficial wound infection. The patients with the 2 cases of bone nonunion refused to undergo another operation (shown in Figure 6). At present, extracorporeal shock wave treatment is given to patients with bone nonunion. The symptoms of wound infection disappeared after wound dressing changes, and oral antibiotics were administered. Complications occurred in 2 patients in group B, and 1 patient with internal fixation cut-out. The internal fixation instrumentation was removed (reoperation). There was 1 case of superficial infection in the wound, and the symptoms disappeared after the oral administration of antibiotics. No deep infections requiring reoperation were found in either group. Overall, between group A and group B, postoperative complications had no noticeable differences (*p* > 0.05).

## 4. Discussion

### 4.1. Surgical Technique

Subtrochanteric fractures make up 4% to 18% of all proximal third femur fractures [22, 23]. At present, these fractures are challenging for clinicians to treat, and improper treatment can lead to severe complications. The most common complications are fracture nonunion, varus deformities, and internal fixation incisions. According to anatomical studies, these fractures mainly occur in the proximal part of the femur from the lesser trochanter to the isthmus, especially in young men and elderly patients. In the clinic, the first-line treatment for long, oblique subtrochanteric fractures of the femur is the corresponding surgical treatment. However, when the operation time is extended, some patients exhibit poor healing, and in severe cases, disability and poor quality of life. Therefore, finding a safe and effective operation is the key to improving the prognoses of patients [24].

In our study, both groups of patients underwent lower limb distractor reduction and rapid assembly in a minimally invasive way. With these techniques, the hip joint can be abducted and adducted at will, which accelerates nail insertion and fracture reduction. The thickest part of the iliac wing is the proximal acetabular nail, and its hardness is high enough to be a perfect fracture reduction fulcrum. Just above the long axis of the patella is the distal connection point, which is parallel to the long axis. The medial and lateral sides of the femur can be extended by the distal extension rod, and the medial and lateral angulation of the fracture can be corrected. Sufficient stability can be provided if the distal sliding structure is deployed to spread the fracture location [25].

### 4.2. Advantages of Wire Cerclage

The key to good results and decreasing the risk of complications is the fracture's near-anatomical reduction and the fracture fragments' optimal location. Furthermore, wire cerclage can provide medial cortical support, which is an important factor in promoting fracture healing and preventing varus deformities and nonunion [8, 26, 27].

More evidence showed that compared with the biological disadvantage of open reduction, the mechanical advantage of preventing varus deformity and restoring medial cortical support is greater [28]. The periosteal vascular supply in the subtrochanteric region is circumferential. Studies have shown that after the use of cerclage wire, vascular supply is conserved [29]. As a result, if the application of the subtrochanteric cerclage wire is helpful for anatomical fracture reduction and stabilization, it is safe and valuable [30]. By providing the medial cortex, the cerclage wire sustains and accelerates the healing of the medial cortex. According to Hoskins et al. [4] and Kennedy et al. [10], cerclage cables/wires are beneficial for treating this type of fracture. The subtrochanteric fractures' revision rates reported in other studies are similar to those observed in our study. Karayiannis et al. [9] found by a retrospective analysis that the fixation of subtrochanteric fractures can be increased by cerclage/wire. Underlying advantages contained an improvement in the reduction quality.

### 4.3. Analysis of Main Results

In the group without steel wire ring ligation, we performed closed reduction or clamp-assisted reduction and removed the clamp after the intramedullary nail was placed. The fracture pieces of 4 patients were displaced again by an average of 3–5 mm. This occurrence may be the reason that the fractures took a long time to exhibit healing radiologically. Juan Mingo-Robinet et al. performed a study on the treatment of elderly subtrochanteric fractures with minimally invasive clamp reduction without cerclage and intramedullary nails and found that the fractures were fixed with intramedullary nails without cerclage. The risk of infection would be increased because the cerclage wire needed more soft tissue dissection, which prolonged the operation time and needed more hardware [31].

Patients who received cerclage had longer hospital stays in this study than those who did not. Also, this may be because of the need to cut and reset to allow the looped cables/wires to pass through. In this process, a larger incision would be needed, which can lead to increased blood loss and postoperative pain.

### 4.4. Analysis of the Complications

In group A, there were 2 patients with fracture nonunion, one of which was caused by the redisplacement of the fracture block after the operation, which led to the enlargement of the fracture space, and the patient refused to undergo reoperation because of family financial difficulties. Another patient had a comminuted fracture, the proximal fracture healed well, and the distal fracture showed nonunion 1 year after the operation. Because the patient complained of no special symptoms and refused surgical treatment, the patient was currently receiving extracorporeal shock wave treatment. In group B, one elderly patient underwent internal fixation cut-out, which may be related to osteoporosis, and hip arthroplasty was performed after the internal fixation was removed [32–38]. One case of superficial wound infection occurred in both groups, which may be related to the extent of damage to the soft tissue of the wound. All patients healed well after antibiotics and dressing change, and no deep infection occurred.

### 4.5. Limitations of the Study

We recognize that this study has essential deficiencies. Because of the retrospective characteristic, this study could not regulate the loss to follow-up rate, and complications may have been underreported due to patients presenting to other hospitals. Moreover, the patient population was not homogenous with respect to age, sex, or the type of trauma causing the fracture. The sample sizes of the two groups were relatively small. Hence, we are going to launch multicenter and proactive research with a larger sample size to verify the findings of this study.

## 5. Conclusions

This study showed that the vital point to the successful treatment of unstable femoral subtrochanteric fractures was the anatomical reduction, and the use of cerclage wires may improve the fracture reduction effect. A remarkable distinction was observed between the two groups in the operation time (*p* < 0.05), quality of reduction (*p* < 0.05), and union time (*p* < 0.05). Reduction and wire cerclage are cut to give medial support for the anatomical reduction, which has a positive effect on fracture healing.

Our results indicated that the benefits of using a cerclage wire for fracture reduction outweigh the risks, although many subtrochanteric fractures may be successfully treated with a single indirect reduction.

## Figures and Tables

**Table 1:** Baseline characteristics and clinical outcomes of Group A and Group B.

	Group A (*n* = 41), %	Group B (*n* = 27), %	*p* value
Sex (male)	30 (73.17)	19 (70.37)	0.80
Age (y)	58.98 (31–83)	57.04 (30–83)	0.65
Seinsheimer classification			0.76
IIB	13 (31.71)	8 (29.63)	1.00
IIC	2 (4.87)	1 (3.70)	1.00
IIIA	11 (26.83)	4 (14.81)	0.37
IIIB	1 (2.44)	2 (7.41)	0.56
IV	9 (21.95)	7 (25.93)	0.77
V	5 (12.20)	5 (18.52)	0.50
Quality of reduction			0.03
Good	24 (58.54)	22 (81.48)	
Acceptable	12 (29.27)	5 (18.52)	
Poor	5 (12.20)	0	
Implant type			0.94
Gamma3	12 (29.27)	9 (33.33)	
InterTan	8 (19.51)	5 (18.52)	
PFN	21 (51.22)	13 (48.15)	
Operation time (min)	102.05 ± 29.04	124.01 ± 35.28	0.04
Estimated-blood-loss (ml)	145.73 ± 75.50	172.59 ± 83.79	0.12
Complication rate	3 (7.32)	2 (7.41)	0.86
Nonunion	2	0	
Infection	1	1	
Cut-out	0	1	
TAD (cm)	20.34 ± 4.38	21.11 ± 3.21	0.33
Varus/valgus	126.46 ± 2.89	127.44 ± 2.08	0.83
Follow-up time (months)	17.58 ± 4.58	19.44 ± 5.53	0.16
Day of stay (days)	8.22 ± 1.97	9.52 ± 2.01	0.08
Union time (months)	4.82 ± 0.89	3.56 ± 0.64	0.01
HHS	76.29 ± 9.27	75.59 ± 8.88	0.64

## Data Availability

The simulation experiment data used to support the findings of this study are available from the corresponding author upon request.
